# 

**DOI:** 10.1192/bjb.2020.145

**Published:** 2021-04

**Authors:** Kamaldeep Bhui

**Affiliations:** Professor of Psychiatry at the University of Oxford, and honorary consultant psychiatrist at the East London NHS Foundation Trust, UK. Email: kam.bhui@psych.ox.ac.uk

The contributors to this refreshing, courageous and remarkable book break the silence about young people living with parental mental illness and the distress they and their immediate and future families experience. The central theme is to enable conversations about difficult experiences of parents and children. Airing personal reflections and experiences risks rejection, stigma, hurt and isolation. Traumatic experiences are often associated with avoidance of reminders and a desire to imagine the world to be different and just, rather than the more abrupt reality of moral fractures in a world where fragile and vulnerable minds are overwhelmed. Parents wanting so much for their children find themselves unable to live up to their own expectations, and at the same time lament that they may be hurting their children inadvertently, just by living with mental illness. Yet, these sentiments are not easily realised, and much time is spent by parents and children in evading or not knowing how the other feels and sees the world, including in their relationships.

The contributors include young people who were raised by people with mental illnesses, some of whom have gone on to become parents and enter the professions that work with children not dissimilar to their past selves. Additionally, in the book, parents offer commentary and children in return reflect on their parents’ dilemmas and distress. Although it is painful to confront such realties, all contributors are optimistic, full of gratitude, being mindful of how the world looking back seems so different from how it felt in the moment.

Through a network of conversations, observations, reflections and counter-reflections, the editors provide a form of scaffolding that helps. They create and display the very process that needs to take place between parents and children facing fears and isolation that come with parental mental illnesses. They reveal how intimate and important sentiments surfaced and were discussed in a reflective fashion by families to arrive at what seems like a more settled and peaceful coexistence, an appreciation of each other's strengths, and ultimately the enduring respect and love that evolve as they show they care so much for each other. That they matter more than they realise.

There is much here for professionals, parents and children, as well as commissioners and policy makers. The first-person accounts are invaluable, articulate and perhaps even a little refined and polite, having been worked at for some time. This makes it easier to hear about some very harrowing moments in the lives of those bearing witness to and living with the silent struggles. I do hope that those affected will read the book and discover they need not be alone and have much to give and have more courage than they might realise.

